# Anti-Cancer Properties of *Heterotrigona itama* sp. Honey Via Induction of Apoptosis in Malignant Glioma Cells

**DOI:** 10.21315/mjms2019.26.2.4

**Published:** 2019-04-30

**Authors:** Farizan Ahmad, Priatharsine Seerangan, Mohd Zulkifli Mustafa, Zul Faizuddin Osman, Jafri Malin Abdullah, Zamzuri Idris

**Affiliations:** 1 Department of Neurosciences, School of Medical Sciences, Universiti Sains Malaysia, Kubang Kerian, Kelantan, Malaysia; 2 Human Genome Centre, School of Medical Sciences, Universiti Sains Malaysia, Kubang Kerian, Kelantan, Malaysia; 3 School of Dental Sciences, Universiti Sains Malaysia, Kubang Kerian, Kelantan, Malaysia; 4 Centre for Neuroscience Services and Research, Universiti Sains Malaysia, Kubang Kerian, Kelantan, Malaysia; 5 Hospital Universiti Sains Malaysia (HUSM), Universiti Sains Malaysia, Kubang Kerian, Kelantan, Malaysia

**Keywords:** malignant glioma, stingless bee, Heterotrigona itama sp., apoptosis, anti-cancer

## Abstract

**Background:**

There has been increasing evidence showing that stingless bee honey exhibits anti-oxidant, anti-inflammatory and anti-cancer properties. Pharmacologically-active components in honey such as flavonoids and phenolic constituents are known to contribute to its medicinal benefits. To the best of our knowledge, this is the first study on evaluating anti-cancer effects of locally-produced Malaysian stingless bee honey from *Heterotrigona itama* sp. on malignant glioma cells.

**Methods:**

Proliferation and apoptosis studies of U-87 MG cells following stingless bee honey treatment were carried out using MTS assay and acridine orange/propidium iodide dual staining, respectively.

**Results:**

Results demonstrated time and dose-dependent cytotoxicity using 0.625%, 1.25% and 10% stingless bee honey (*P* < 0.05). IC_50_ values were calculated using cells treated with 10% stingless bee honey. It was also observed that 10% stingless bee honey induced nuclear shrinkage, chromatin condensation and nucleus fragmentation, indicating that cellular changes were consistent with the apoptotic characteristics of the cells.

**Conclusion:**

These data provide a good basis for further evaluation of the medicinal properties of stingless bee honey from *Heterotrigona itama* sp. This source of honey may serve as a potential therapy for malignant glioma.

## Introduction

Malignant glioma is the most common histological type of brain tumour, characterised by aggressive growth, rapid progression and poor prognosis. The annual incidences of malignant gliomas are increasing worldwide and in Malaysia (1). Despite various improvements in the existing treatment approaches, the prognosis of malignant glioma patients remains poor, with a mean survival of only 14.6 months (2). The treatment of these tumours has been particularly challenging due to the fact that the malignant cells are infiltrative in nature and often lead to tumour recurrence.

Currently, researchers are seeking for new substances that may reduce the viability of cancer cells, slow the tumour growth, and extend patient life expectancy. Therefore, there has been increased interest in natural products for their possible role in preventing or treating cancer. Among the meliponinicultural products with functionality characteristics (anti-bacterial, anti-inflammatory, anti-viral), stingless bee honey has been widely studied in various cultured cell lines (3, 4). Stingless bees produce honey which is stored in propolis-rich cerumen pots. Compounds such as polyphenols (phenolic acids, flavonoids and their derivatives), terpenes, steroids and amino acids are of particular interest, as they are considered important in traditional medicine (5). A previous study by Jaganathan and colleagues reported strong cytotoxicity of honey in U-87 MG cell line. They suggested a possible connection between the observed cytotoxicity to the polyphenols content of the honey (6). The paper also proposed that polyphenols in honey such as caffeic acid, caffeic acid phenyl ester, chrysin, galangin, quercetin, kaempferol, acacetin, pinocembrin, pinobanksin and apigenin may be responsible for the anti-proliferative activity observed. In addition to that, other biochemical compounds found in honey such as the chrysin can be used to prevent cancer in a manner similar to the action of a breast cancer drug (anastrozole) (7).

Stingless bee honey comprises of sugar and water (95% of the dry weight) along with numerous other compounds such as organic acids, proteins, amino acids, minerals, polyphenols, vitamins (ascorbic acid) and aroma compounds. Pharmacologically active components, especially flavonoids and phenolic constituents are thought to contribute towards the medicinal benefits of these stingless bee honey (8).

Recently, a project named reinventing honey quality (RHQ) has been introduced to empower the growth of stingless bee industry in Malaysia. This project has a direct impact on the quality of stingless bee honey products, ensuring hygienic practices in a systematically organised farm (9). Therefore, this study aims to investigate the anti-cancer properties of stingless bee honey from *Heterotrigona itama* sp. in malignant glioma cells, U-87.

## Methods

### Cell Culture

Human malignant glioma cell line U-87 MG (ATCC^®^ HTB-14™) was obtained from the American tissue culture collection (ATCC) and grown in Dulbecco's modified eagle medium (DMEM) (Sigma Aldrich) media supplemented with 10% fetal bovine serum (FBS, Gibco) and 1% penicillin and streptomycin (Gibco). Cells were incubated at 37 °C with 5% CO_2_.

### Collection and Preparation of Heterotrigona itama sp. Honey

To ensure quality and standardisation of stingless bee honey sample, *Heterotrigona itama* sp. bees were grown in the artificial Mustafa- Hive (Meliponiculture Using Split-able Throne within Air-jacketed palace For Amplification-Hive) which facilitates colony expansion and health screening, while simultaneously providing a habitat that can be adapted to changing climatic conditions. The Mustafa-Hive effectively promotes a more hygienic honey production practice via in-door harvest approach from systematically organised farms localised system. The honey was collected under sterile condition. Working concentrations of the honey were prepared prior to each assay by diluting the honey with DMEM media to final concentrations ranging 0.16%–10% (v/v). The working solution of honey was then filtered using 0.45 μL sterile filter (millipore).

### Treatment of Cells Using Heterotrigona itama sp. Honey

The amount of 0.5x10^5^ cells/mL were seeded into three sets of 96-well plates. Plates were then incubated overnight at 37 °C with 5% CO_2_. On the following day, media were removed and rinsed. Then, 100 μL of new assay media was added. The cultured cells were treated with *Heterotrigona itama* sp. honey in triplicate wells for each condition, at a final concentration of 0.16%, 0.31%, 0.63%, 1.25%, 2.5%, 5% and 10% (solvent control) μg/mL, in which these concentrations were achieved by means of serial dilution. The plates were incubated for 24, 48 and 72 h. Upon each time interval, media from the plates were discarded and cells were rinsed using 100 μL of PBS. Fresh media of 90 μL was added into the wells followed by 10 μL of 3-(4,5-dimethythiazol-2-yl)-5-(3-carboxymethoxyphenyl)-2-(4-sulfophenyl)-2Htetrazolium (MTS) reagent (Promega). The plates were covered with aluminum foils and incubated at 37 °C for 2 h. Upon incubation period, the absorbance was measured at a wavelength of 570 nm using a microplate reader (VersaMax). The cytotoxic effect of *Heterotrigona itama* sp. honey was assessed by calculating the percentage of cell viability compared to negative controls. IC_50_ values were determined from the plotted graph. The optical density (OD) and mean cytotoxicity was calculated as follows:

% of cytotoxicity=(A sample-Ab)/(Ac-Ab)

A sample = Absorbance of sample
Ab = Absorbance of blank
Ac = Absorbance of control

### Morphological Analysis of Cells Following Treatment

Cells were seeded in 6-well plates at 5 × 10^5^ cells/mL and incubated overnight at 37 °C with 5% CO_2_. The cells were treated with 2% and 10% concentration of the stingless bee honey for 72 h. Upon treatment, morphological changes were examined using light microscopy (Carl Zeiss, Germany).

### Apoptosis Analysis Using Acridine Orange/Propidium Iodide Staining

Dual staining technique using a cell-permeable DNA-binding dye Acridine Orange (AO) in combination with membrane impermeable DNA-binding dye propidium iodide (PI) was carried out. AO/PI excites green and orange fluorescence when bound to DNA. Early apoptotic and live cells would take up AO dye and emit green fluorescence, while late apoptosis and necrosis cells would take up PI dye and emit red fluorescence. Characteristics of apoptotic bodies include plasma membrane blebbing and chromatin condensation. A total of 100 cells were counted and the apoptotic index was calculated using the following formula:

Apoptotic index=(Number of apoptotic cells/total number of cells)×100

Upon IC_50_ honey concentration treatment, cells were dislodged into bullets. A mixture of AO and PI was prepared as the stock solution. The working solution was prepared by means of adding 1 μL of the AO/PI stock solution into a fresh bullet containing 100 μL of phosphate-buffered saline (PBS). Then, 1:1 ratio of the fluorescent dye (100 μL) and cells (100 μL) were mixed in the new bullet and left at room temperature for 5 min in the dark before analysis. A slide was prepared immediately where live, necrotic and apoptotic cells were counted. For fixed cells, cells were grown in a 4-chambers slide and allowed for overnight attachment before treatment with IC_50_ honey concentration for 72 h. Upon treatment, cells were washed using three times with PBS for 5 min. Cells were fixed using cold methanol for 20 min and another three PBS washes for 5 min. Cells were permeabilised using Triton-X for 5 min and then washed with PBS. AO/PI staining was done as mentioned above and slides were kept in the fridge at 4 °C for future viewing and analysis. The apoptotic index was further calculated.

### Statistical Analysis

Results obtained were presented along with the mean and standard deviation after three independent experiments were performed. Repeated measure analysis of variance (ANOVA) was performed to help determine the significance of inhibition of cell proliferation following honey treatment. Grouped cells were treated for 24 h, 48 h and 72 h, respectively. The significance value cut-off was set at *P* < 0.05.

## Results

### Cytotoxic Analysis of Heterotrigona itama sp. Honey on Malignant Glioma Cells

**Figure 1:**
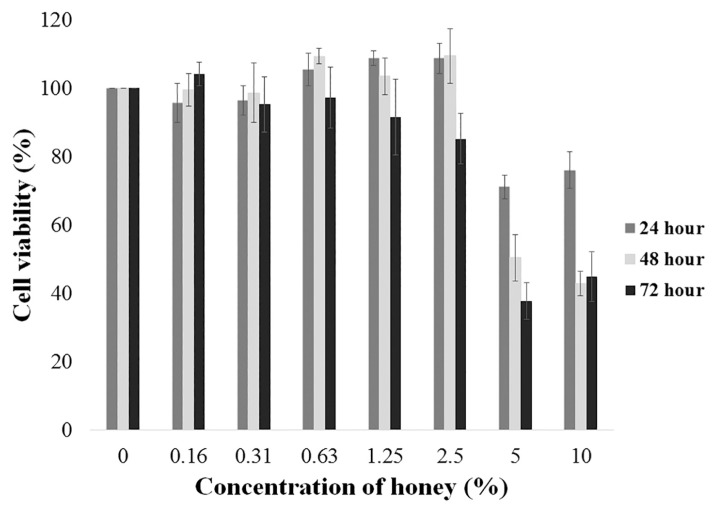
Cytotoxic effect in U-87 MG cells upon various concentration of *Heterotrigona itama* sp. honey at 24 h, 48 h and 72 h. The cells were treated with various concentrations of honey up to 10% for up to 72 h. Each value represents the mean SD from three independent experiments. Best IC_50_ value for U-87 MG cells was observed in cells treated with 10% of honey.

The data revealed that the highest cytotoxic effect was achieved after 72 h of honey treatment. The honey did not produce any considerable cell inhibitory effect at 24 h and 48 h. In fact, the cells were observed to be proliferating with minute cell death. The obtained results were therefore concluded to be time and dose-dependent. The inhibition of 50% cell viability (IC_50_) for U-87 MG cells was obtained at 10% of honey treatment (Figure 1) with standard deviation (SD) from three independent experiments. The significant inhibition of cells proliferation treated with 10% of honey was observed between 24 h and 48 h post-treatment (*P* = 0.018) and between 48 h and 72 h post-treatment (*P* = 0.028). A statistical test was carried out using repeated measure ANOVA (Figure 1).

### Morphological Changes of U-87 MG Cells

**Figure 2:**
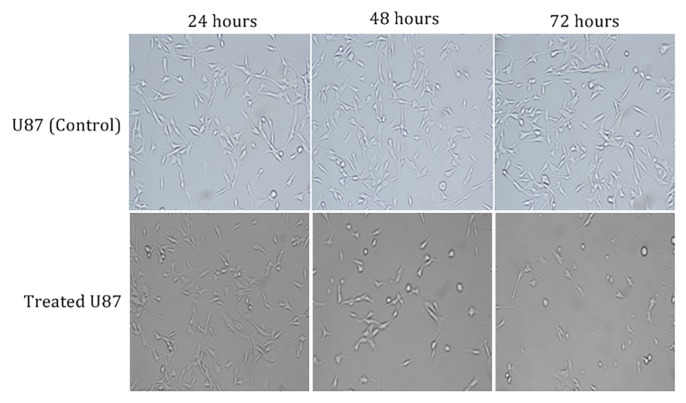
Inverted microscopy images of untreated and treated groups of U-87 MG cells with 10% concentration of *Heterotrigona itama* sp. honey at magnification of 10x. Inhibition of cell proliferation rate were observed at 48 h and 72 h post treatment

The morphology of U-87 MG cells treated with *Heterotrigona itama* sp honey at concentrations of 0.625%, 1.25% and 10% for 24 h, 48 h and 72 h was analysed. Untreated cells showed increased proliferation up to 72 h of incubation, apart from a few cell detachment indicative of normal cell death. However, upon treatment of honey, cells started to shrink and appeared rod-like in shape. After 72 h, most cells were seen to round up and lose adhesion, confirming cell death by apoptosis. The highest number of cell death was observed in U-87 MG cells treated with 10% of honey. The morphological changes of U-87 MG cell lines after treatment with *Heterotrigona itama* sp. honey are shown in Figure 2.

### Apoptotic Analysis of U-87 MG Cells

**Figure 3:**
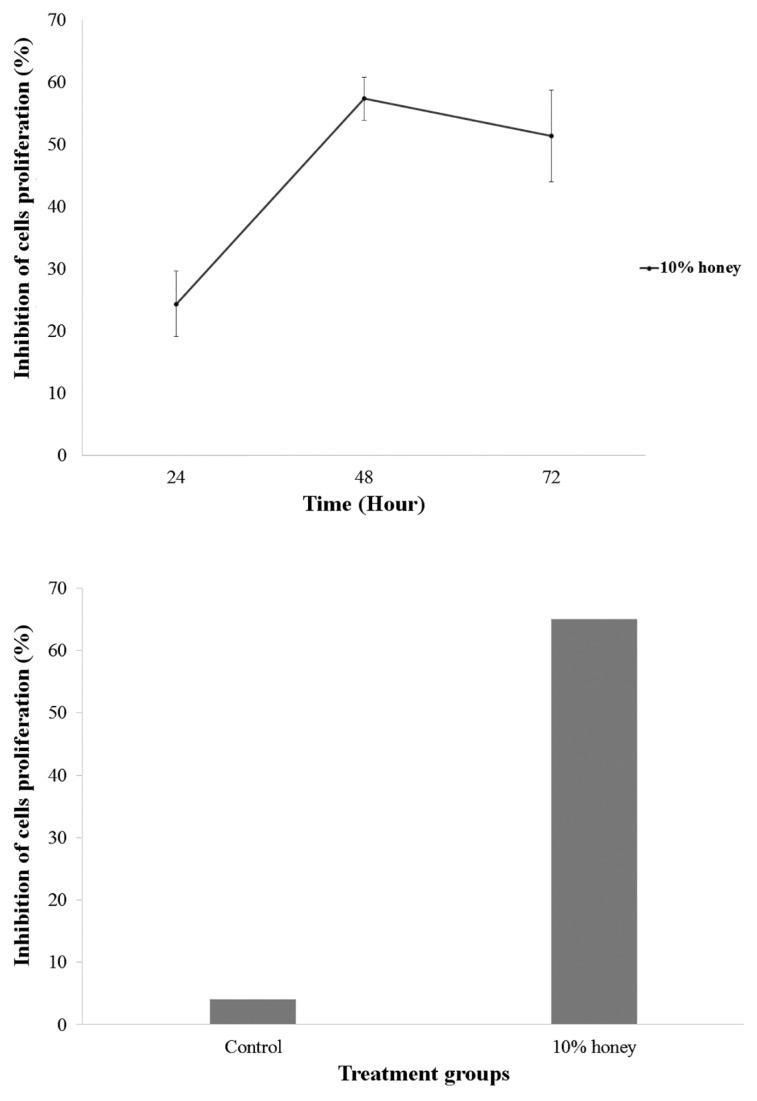
Inhibition rate of U-87 MG cell proliferation following honey treatment at 10% concentration. (A) Inhibition of cells proliferation at 24 h, 48 h and 72 h; (B) Inhibition of cells proliferation at 72 h for untreated (control) and treated group (10% honey)

**Figure 4:**
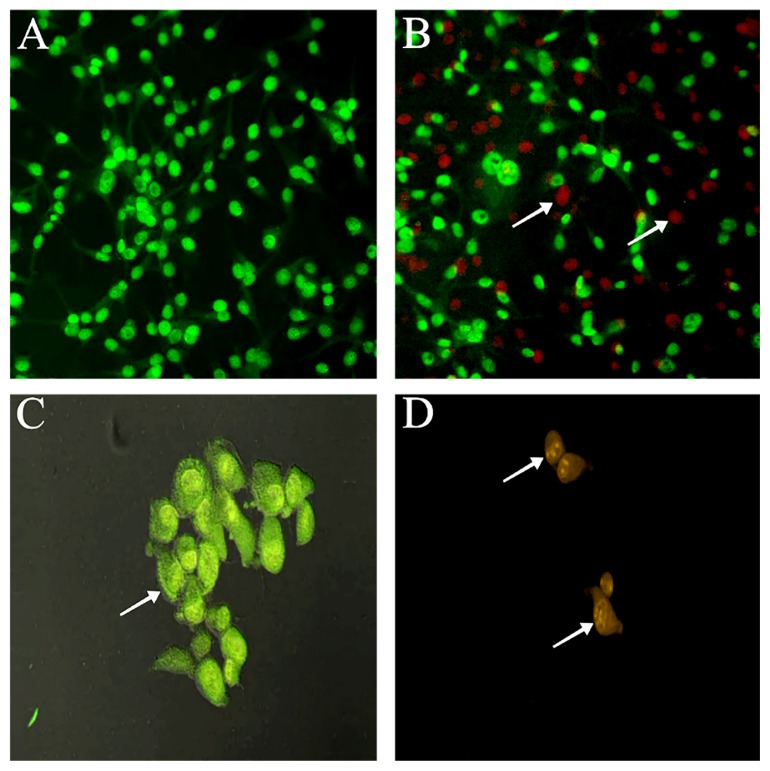
Fluorescent micrograph of AO/PI double-stained U-87 MG cells treated with 10% *Heterotrigona itama* sp. honey for 72 h. Untreated U-87 MG (A) and showed normal structure of the cells. Whereas in the treatment group, a significant number of U-87 MG cells underwent apoptosis were detected as shown by the orange to red coloured cells (arrow, B). The images were taken at 100 magnification. Nuclei of viable cells are green with organised structure, whereas nuclei of early apoptotic cells are bright green as in U-87 MG (arrow, C). Whereas late apoptotic cells display bright orange to red with condensed chromatin in U-87 MG (arrow, D). The images were taken at 400x magnification

In order to determine the type of cell death following honey treatment on u-87 MG cells, dual staining technique using fluorescent DNA binding dyes AO/PI was carried out. AO is a cel-lpermeable dye, fluorescing bright green for all nucleated cells whilst PI dye is only permeable to non-viable cells and gives out orange/red colour. Therefore, live cells are bright green in colour with an undisturbed membrane, early apoptotic cells contain green nuclei with chromatin condensation, and late apoptotic cells fluoresce orange/red. After 72 h, an increase in the percentage of apoptotic cells was detected compared to the control (untreated). Results obtained showed a dose-dependent increase in the percentage of apoptotic cells with a significant decrease in the percentage of live U-87 MG cells (Figure 3). Images of the cells from AO/PI staining are shown in Figure 4.

## Discussion

There has been growing evidence showing that honey, in general, contains anti-bacterial and wound healing (10), anti-oxidant (11, 12), anti-inflammatory (13, 14) and anti-cancer (15, 16, 17, 18) properties. However, the biological activities and clinical potential of stingless bee honey are yet to be fully elucidated (19).

Current knowledge on stingless bee honey is limited to the anti-microbial and anti-proliferative activities of *Tetragonula laeviceps* sp. reported in Thailand (20, 21) and *Melipona scutellaris* sp. from Brazil (22). The gap of knowledge on stingless bee honey potential has prompted us to study the anti-cancer properties of *Heterotrigona itama* sp. stingless bee honey, which are locally found in Kelantan, Malaysia.

The ability of honey to inhibit cancer cell growth depends on the bee product source, species and also type of cancer cell lines used. This is in agreement with a study conducted in Indonesia, in which Indonesian stingless bee honey products (*Timia incisa, T. apicalis, T. fusco-balteata* and *T. fuscibasis*) were found to inhibit the growth of five different types of human cancer cell lines (HepG2, SW620, ChaGo-1, KAT0-3 and BT474) with various sensitivities (23). Overall, their results revealed that KAT0-3 cells showed the least cytotoxicity upon stingless bee honey treatment and failed to reach 50% inhibition, whereas, HepG2 cells showed the maximum sensitivity. SW620 cells reportedly showed no significant cell inhibition upon treatment with all five stingless bee species honey extract (23). In another study, the cytotoxicity activity of *Tetragonula laeviceps* sp. honey against various malignant cell lines was tested in BT474, Chago, HepG2, KAT0-3 and SW620 cells.

In this study, *Heterotrigona itama* sp. honey was found to exert cell proliferation inhibitory effect in U-87 MG cells in a time and dose-dependent manner, coherent to Kustiawan and colleagues’ studies (23). Cytostatic cells were inhibited from proliferating and therefore inhibited in growth (24). However, the maximum cell death was observed at 10% concentration of honey used.

In a previous study, the anti-proliferative effect of stingless bee―Tubi (*Scaptotrigona* sp.) product obtained from Serra Corda region, Brazil, showed comparable differences upon treatment in two types of glioblastoma cell lines, U251 and U343. Though both cells presented a dose and time-dependent anti-proliferative effects, U343 cells were found to be more sensitive than U251 cells. The study revealed the possibility of p53 status (wild type/mutant) association to be causal for the differences in cell viability inhibition in both cell lines used (3).

Morphological changes in both U-87 MG cells exposed to several different concentrations of honey corroborates with our MTS assay results. Results revealed an inhibitory effect only after 72 h, whilst during the time interval of 24 h and 48 h, there was observable cell proliferation. This suggests that the inhibitory effects of the honey in the malignant glioma cell lines could only be achieved significantly after 72 h. Observed morphological changes in the treated cells include loss of cell adhesion, cell shrinkage, increased number of rounded cells, detachments and compromised cell density. These characteristics are often associated with early cell death process, given that vital cell proliferation is dependent on cell membrane integrity. Cell rounding and loss of cell extension were observed mostly at 72 h in cells exhibiting typical characteristics of apoptosis. The control group of cells revealed confluent cell population with less rounded and floating dead cells.

Using AO/PI staining, it was observed that changes were typical to cells undergoing apoptosis such as apoptotic bodies, membrane blebbing and cell detachments. Following the dual staining technique, live cells and apoptotic cells were distinguished and scored to build the apoptotic index (Figure 3). When observed under the fluorescence microscope, chromatin condensation was visible. U-87 MG cells with stained AO/PI for 72 h without honey treatment revealed a uniform green nucleus indicative of live cells. However, in 10% honey treated cells, early apoptosis was identified with yellow staining indicative of the presence of intercalated AO within the fragmented DNA in the cells. The appearance of orange/reddish stained cells was suggested to be cells undergoing late apoptosis due to the binding of PI to the denatured DNA. Late apoptosis was predominantly observed in U-87 MG cells treated with the honey.

The ability of stingless bee honey to induce apoptosis in malignant glioma cells makes it a potential candidate for the development of a new therapeutic approach to improve the wellbeing of cancer patients. The absence of studies related to stingless bee honey has prompted us to compare our data with other studies which demonstrated cytotoxicity and apoptotic effects of stingless bee propolis. The study used Indian stingless bee propolis against different human malignant cell lines and reported its anti-cancer property. The study also postulated that flavonoids compounds present in the propolis possess anti-oxidant activities and therefore substantiate the anti-cancer activity (25). Though there is currently no physiochemical content analysis on *Heterotrigona itama* sp. honey, stingless bee honeys, in general, contained numerous compounds such as organic acids, proteins, amino acids, minerals, polyphenols, vitamins (ascorbic acid) and aroma compounds (26).

In comparison to normal honey, stingless bee honey exhibits higher moisture content, higher ash content, lower pH, higher acidity and lower diastase activity (27). Reactive oxygen species (ROS) often present in minimal amount. A high amount of ROS can result in oxidative stress in cells. Therefore, an agent with the ability to neutralise an excess amount of ROS is known to contain anti-oxidant capacity (28, 29). It is possible that the anti-cancer activity exhibited by *Heterotrigona itama* sp. honey in U-87 MG cells in this study is mediated by the anti-oxidant capacity resulting from the presence of free radicals scavenging ability of phenolic (benzoic, cinnamic and p-coumaric acid) and flavonoid content of this honey. These components have been previously documented in many cytotoxic and anti-oxidant studies in several malignant cell lines such as K562, HeLa and LNCaP cells (2, 30, 31). Nevertheless, further studies should be carried out in the future to support this hypothesis.

Notably, our study demonstrated the anti-cancer properties of *Heterotrigona itama* sp. honey via the induction of apoptosis in malignant glioma cells. The cytotoxic and morphology analyses data consistently showed that the effects were dose and time-dependent.

## Conclusion

The focus on the honey product of stingless bee *Heterotrigona itama* sp. in the current study is due to its growing distribution and industrial potential in Malaysia. Importantly, this study demonstrated promising anti-cancer activities of the honey via inhibition of cells proliferation and induction of early apoptosis in malignant glioma cell lines. Data from this study may facilitate further investigation to determine the molecular mechanisms involved in the cytotoxicity and apoptosis nature of the honey.
